# Validity and reliability of the Persian version of the short-form child perceptions questionnaire 11–14-year-old children (CPQ11–14)

**DOI:** 10.1186/s12955-022-02017-6

**Published:** 2022-07-22

**Authors:** Tahereh Baherimoghadam, Shahram Hamedani, Navid Naseri, Alireza Ghafoori

**Affiliations:** 1 Department of Orthodontic, School of Dentistry, Shiraz Branch, Islamic Azad University, Shiraz, Iran; 2 Oral and Dental Disease Research Center, School of Dentistry, Shiraz University of Medical Sciences, Shiraz, Iran; 3 Student Research Committee, School of Dentistry, Shiraz University of Medical Sciences, Shiraz, Iran

**Keywords:** Oral health-related quality of life, Child perceptions questionnaire, Reliability, Validity

## Abstract

**Background:**

The Child Perception questionnaire 11–14 (CPQ 11–14) is an efficient tool for assessment of oral health-related quality of life (OHRQoL). This questionnaire has been briefed to 16 items (CPQ11–14 ISF-16) to facilitate its employment. The purpose the present study was to translate CPQ11–14 ISF-16 to Persian language, culturally adapt, and initially evaluate its among adolescent population.

**Material and method:**

The questionnaire has been translated to Persian, then back-translated to English, and finally underwent cultural adaptation and pretesting assessment. It was then filled out by 318 participents (175 grils and 143 boys), with the age of 11 to 14 years resident in Shiraz, Iran. The Persian version of CPQ11–14 ISF-16 along with the Psychosocial Impact of Dental Aesthetics Questionnaire (PIDAQ) and two suggested global questions were administered among participants to assess its criterion validity.

**Results:**

The factor analysis extracted two domains and the factor loading of domains ranged from 0.423 to 0.837. Persian version of CPQ11–14 ISF-16 presented high internal consistency (Cronbach’s alpha = 0.854), and showed excellent criterion validity with PIDAQ (rho = 0.731, *p* < 0.001). There was a statistically significant positive moderate correlation between CPQ11–14 ISF-16 and its factors 1 and 2 and two global questions (first global question: rho = 0.439, *p* < 0.001; second global question: rho = 0.457, *p* = 0.035).

**Conclusion:**

The Persian version of CPQ11–14 ISF-16 has optimal validity and reliability in a general population of 11–14-year-old Persian children.

## Introduction

One of the imperative concepts related to children's health, which can play an effective role in their lives, is the concept of health related quality of life. Health related quality of life is a multidimensional notion that seeks particular aspects such as the physical, mental, and social dimensions based on the concept of health defined by the World Health Organization (WHO).

In addition, is influenced by personal experiences, beliefs, expectations, and feeling [1]. Health related quality of life expresses a personal feeling of physical and mental health and the ability to react to various aspects of the physical and social environment [2]. Health related quality of life is an important concept in dental health research. [3]

One of the questionnaire that deals with the relationship between oral health quality and children's life is the Child Oral Health Quality of Life (COHQoL) questionnaire [4]. This questionnaire is a set of multidimensional measurement comparisons for the possible negative effects of oral and orofacial problems on children aged 6 to 14 years and their families. One of the main components of this questionnaire is Child Perceptions Questionnaire aged 11 to 14 (CPQ 11–14). The CPQ Questionnaire is a 37-item questionnaire obtained from Oral Health Quality of Life (OHRQoL) and designed in 2002 in Canada [5]. These 37 questions embrace the biopsychosocial model of health and are organized into four health sections including oral symptoms (6 questions), functional limitations (9 questions) emotional well-being (9 questions), and social well-being (13 questions). For use in clinical population-based health study, CPQ 11–14 has been shortened to 16 items (CPQ11-14 ISF-16) with satisfactory psychometric properties [6–8]

The questionnaire also includes two global questions about the child's oral health and the extent to which the children's oral and orofacial condition affects their general health [5]. Before employment of questionnaires in different cultures and countries, the fulfillment of a translation and validation process that accounts for the cultural and social aspect of the new region is indispensable. [9, 10] CPQ has been translated to several languages so far and its validity and reliability have been previously confirmed. [2, 11–24] To study the CPQ questionnaire in the Iranian population and its use for Iranian children with various oral disorders and malocclusion, it is crucial to translate this questionnaire into Persian and subsequently appraise it in terms of culture and validity.

This study was conducted to assess the validity and reliability of the Persian version of CPQ11–14 ISF-16 and its cultural adaptation.

## Methods and materials

This cross-sectional study has been performed during 2020–2022 at Shiraz, Iran, being one of the biggest cities in South of Iran with Persian population. The study recruited 318 adolescents aged 11 to 14 living in Shiraz, Iran with an average age of 12.08 ± 1.76. Bonett’s formula was applied to determine the sample size. Bonett and Wright proposed a sample size formula for estimating correlations with desired precision and power [25]. A random cluster sampling method was used in this study. We chose 20 schools randomly with the aim of equal presentation of different administrative regions. The classes of students attending grade six to nine were selected as sampling unit (clusters), then we randomly selected participants among these clusters according to sample size.

The following exclusion criteria were defined for this study:

- People with mental and physical disabilities, and psychological disorders
- People with carious teeth with cavities
- People with tooth fracture, severe or moderate fluorosis, or with color spots
- People with positive history of orthodontic treatment
- People who have had cosmetic treatments (laminate, etc.) on their anterior teeth

The original English short version of the CPQ11–14 ISF-16 questionnaire has 16 questions [5]. This version includes four dimensions including oral symptom, functional limitation, emotional well-being, and social well-being. The questions are about the incidents related to the condition of the mouth and jaw in the last three months. The answer options to each of the questions include 0: Never, 1: Rarely, 2: Sometimes, 3: Often, and 4: Every day.

### Primary translation

With the purpose of initial translation of the questionnaire, two fluent English-speaking dentists (with Persian as their mother language), separately and independently, translated the original version of the questionnaire into Persian. After discussion, these two translated copies were turned into a single questionnaire.

### Back translation

In the next step, for back translation, two individuals (with PhD in English translation) who were fluent in English and Persian and familiar with the concept of quality of life translated the Persian version into English. Then, this Persian translation was compared with the original version and all the detected errors were corrected. Accordingly, the Persian version number I of CPQ11–14 ISF-16 was prepared.

### Cross-cultural adaptation

In order to evaluate the qualitative content of this translation, four experts, including two orthodontists, a specialist in oral health and social dentistry, and a person fluent in Persian writing, appraised the Persian Version No. I of the CPQ11–14 ISF-16 and presented their opinions in terms of accuracy, simplicity of the text, Persian grammar, the use of appropriate words and the assignment of words in their correct place. They did the necessary cultural adaptation in the CPQ11–14 ISF-16, and subsequently the Persian version number II of the CPQ11–14 ISF-16 was prepared. The questions number #3, #15, #16 needed slight changes to be more meaningful in Persian; for instance, bad breath does have no specific meaning in Persian and we had to select clearer word which can show the exact meaning of this term in Persian. Before starting the main study, in order to evaluate the potential impediments and assess any conceptual and contextual hindrance, this questionnaire was completed by 20 individuals who referred to the Dental School with age range of 11–14 years old. In order to determine any ambiguity in filling out the questionnaire, an informed and calibrated person was consistently present with the participants at the time of completing the questionnaire and attempted to record any proclaimed objections. After conducting a preliminary study and correcting the existing problems at this stage, the questionnaire was distributed again among 10 other people. After ensuring the translation and fluency of the text, the final Persian version of the CPQ11–14 ISF-16 was finally established. (Table 1).

**Table 1:**
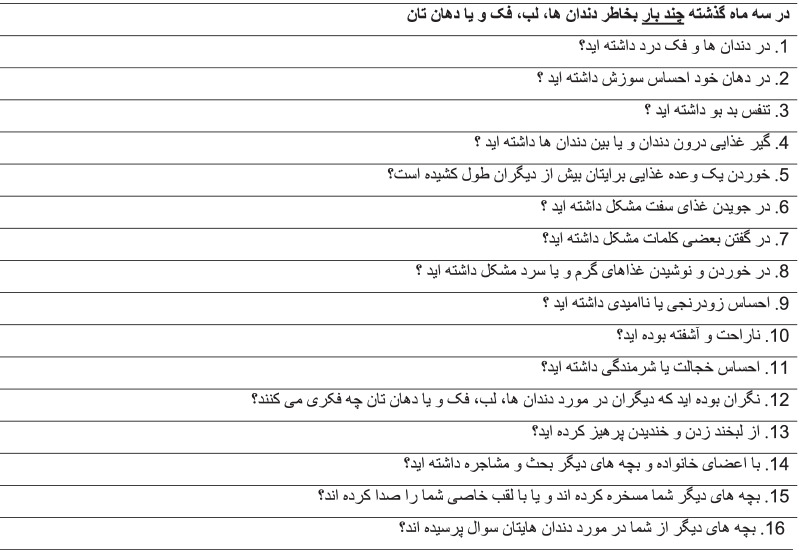
The Persian version of CPQ11–14 ISF-16

To evaluate the validity of the translated version of the CPQ11-1 questionnaire, the Persian version of the Psychosocial Impact of Dental Aesthetics Questionnaire (PIDAQ) questionnaire was used, whose validity and reliability have been previously assessed and reported [26]. In addition, two general questions about the dental and maxillofacial condition and its impact on a person's life were introduced. These questions included:

1. How do you assess the general health of your jaw, mouth, lips, and teeth?
2. In general, how much the condition of your jaw, mouth, lips, and teeth have affected your life?

The teachers of selected classes were instructed about the aims and the process of this study among students. They sent detailed description of the study to parents and asked them for written informed consent if they permitted their child to attend the study. Of 413 parents who received this request for written informed consent, 386 parents sent positive answers. Regarding exclusion criteria, 44 students, and because of uncompleted questionnaire, 24 students were excluded from the study. However; the number of participants was 20% more than sample size which was determined by using Bonnet’s Formula. The students completed the questionnaires in their school classrooms in person; they were asked not to write their names on the questionnaire.

### Statistical Analysis

The Statistical Package for Social Science (SPSS version 18.0, Chicago, IL, USA) was used for data analysis. Distribution of variables was evaluated by Kolmogorov Smirnov method. Exploratory Factor Analysis and Varimax rotation to assess construct validity were applied in this study. Kaiser–Meyer–Olkin measure of sampling adequacy was performed before factor analysis to determine variables capacity for including in factor analysis. Internal consistency of Persian version of CPQ11–14 ISF-16 was tested using Cronbach’s alpha coefficients for the subscales. Criterion validity was tested by comparing total score of CPQ11–14 ISF-16 and scores of its dimensions with PIDAQ and two global questions via one-way analysis of variance (ANOVA) and the Kruskal Wallis test. Test–retest reliability was evaluated with two-way random effects model for 30 randomly selected subjects (15 boys and 15 girls) who responded to the questionnaire a second time after two weeks. This model is suitable for appraisal of rater-based clinical assessment methods, which are designed for routine clinical use by any clinicians with particular characteristics (years of experience for instance) in the reliability studies. In other words, 2-way random-effects model is employed to generalize the reliability results to any raters who hold the same individuality as the selected raters in the reliability study.

The Ethics Committee of Shiraz Azad Dental School has approved the present study (No.48486, 2020).

## Results

318 participants completed the questionnaire correctly. The demographic information is tabulated in Table 2.

**Table 2:** Demographic information of participants

Gender	Girls	Boys	Total
Number	175 (55%)	143 (45%)	318
Mean age ± SD	11.34 ± 2.1	12.78 ± 0.21	12.08 ± 1.76

### Construct Validity

The Kaiser–Meyer–Olkin sample adequacy test showed the amount of overlapping variance across the items in the analysis to be 0.789. The Bartlett's test of sphericity was statistically significant (P = 0.005).These results indicated the adequacy of the sample size for factor analysis. The results of factor analysis of the Persian version of the CPQ11–14 ISF-16 showed two factors that explain a total of 48.711% variance. Questions 1 to 8 make up Factor 1(Well-being), Factor 2 (Symptom and function) includes questions 9 to 16. (Table 3).

**Table 3:** Factor loadings of the CPQ11–14 ISF-16 item scale scores after principal component analysis and orthogonal rotation

	Components		
	Symptom and function	Well-being	Cronbach’s Alpha if Item Deleted
CPQ1	0.026	0.770^**^	0.846
CPQ 2	0.273	0.649^**^	0.842
CPQ 3	0.070	0.576^**^	0.852
CPQ 4	− 0.068	0.703^**^	0.854
CPQ 5	0.219	0.709^**^	0.841
CPQ 6	0.241	0.789^**^	0.837
CPQ 7	0.146	0.423^*^	0.853
CPQ 8	0.132	0.704^**^	0.844
CPQ 9	0.522^*^	0.303	0.845
CPQ10	0.675^*^	0.371	0.835
CPQ11	0.877^*^	0.151	0.836
CPQ12	0.733^*^	0.037	0.846
CPQ13	0.706^*^	0.122	0.844
CPQ14	0.433^*^	0.042	0.854
CPQ15	0.711^*^	− 0.155	0.852
CPQ16	0.618^*^	0.150	0.847
Variance explained (Initial solution)	5.245	2.549	
% of variance Explained (initial solution)	32.782	15.928	
% of variance explained (rotation solution)	24.974	23.737	
Cumulative % of variance explained (rotation solution)	24.974	48.711	
Cronbach’s alpha	0.838	0.830	

A positive, moderate, and statistically significant correlation has been found between the total scores of the CPQ11–14 ISF-16 scale and its factors and the scores of two global questions. (Tables 4 and 5).

**Table 4:** Subscales and CPQ11–14 ISF-16 scores according to first global question

	CPQ Mean ± SD	Symptom and function Mean ± SD	Well-being Mean ± SD
Overall, would you say that your oral and dental health is….			
Excellent	7.23 ± 8.44	3.45 ± 4.24	3.77 ± 6.53
Very Good	10.26 ± 8.51	6.01 ± 4.68	4.26 ± 4.86
Good	15.43 ± 7.39	7.3 ± 4.28	8.13 ± 5.81
Fair	16.32 ± 10.78	8.86 ± 7.19	7.45 ± 5.17
Poor	23.0 ± 7.07	5.5 ± 2.12	17.5 ± 4.95
Spearman correlation	0.439	0.556	0.404
sig	< 0.001***	< 0.001***	< 0.001***

*P* < 0.05; ** *P* < 0.01; *** *P* < 0.001

**Table 5:** Subscales and CPQ 11–14 scores according to second global question

	CPQ Mean ± SD	Symptom and function Mean ± SD	Well-being Mean ± SD
Overall, how much do your teeth affect your life?			
Very much	9.59 ± 8.97	6.09 ± 5.59	3.5 ± 4.10
A lot	16.33 ± 11.65	7.4 ± 5.49	8.93 ± 7.61
Somewhat	13.69 ± 9.98	6.62 ± 6.2	7.07 ± 5.79
Very little	13.24 ± 7.98	6.38 ± 4.82	6.86 ± 6.61
Not at all	8.5 ± 7.85	4.75 ± 4.30	3.75 ± 4.92
Spearman correlation	0.457	0.570	0437
sig	0.035*	0.048*	0.006*

^*^ *P* < 0.05; ** *P* < 0.01; *** *P* < 0.001

### Reliability

Cronbach's alpha correlation coefficient for internal consistency evaluation was 0.854 and the standard value for Cronbach's alpha correlation coefficient was 0.855. (Table 3) Factor 1 had an acceptable reliability of 0.838 and factor 2 had a reliability coefficient of 0.830. Correlation coefficient of Persian version of PIDAQ questionnaire, global question No.1, global question No.2 were 0.898, 0.981, and 0.789, respectively. The test–retest correlation coefficient was 0.85. Moreover, 15 boys (mean age; 12.31 ± 0.54) and 15 girls (mean age; 11.56 ± 1.13) were randomly selected as the participants for evaluation of test–retest reliability.

### Criterion Validity

There was a statistically significant correlation between CPQ11–14 ISF-16 and its factors 1 and 2 and PIDAQ questionnaire. (Table 6).

**Table 6:** Correlation between CPQ 11–14 scores and its subscales and PIDAQ

	CPQ			
			Symptom and function	Well-being
pidaq	Correlation Coefficient	0.731	0.652	0.661
	Sig	< 0.001***	< 0.001***	< 0.001***

*P* < 0.05; ** *P* < 0.01; *** *P* < 0.001

No Floor and ceiling effects were found in this study since the percentages did not exceed 15%.

## Discussion

Since 1980, several studies have introduced the impact of oral and maxillofacial problems on quality of life [27]. Given that most of the tools used in these studies were in English, they must be translated, adopted, and validated to be feasible and applicable when employed in non-English speaking population [28]. The CPQ11–14 ISF-16 has been broadly used in studies on malocclusion and OHRQoL [29, 30]. This questionnaire has been employed for children with dental and maxillofacial malformations. [5]

The CPQ11–14 ISF-16 is a suitable tool to assess the acuity of children and adolescents regarding the condition of their mouth and jaw. To facilitate the use of this questionnaire in population-based surveys, a short form of this questionnaire with 16 items was proposed while retaining its psychometric properties [6]. In this study, the aim of translating the CPQ11-14 ISF-16 questionnaires into Persian was cultural adaptation and validation in a population of adolescents with malocclusion. It should be noted that since the national language of people in Iran is Farsi and this language is uniformly taught in all cities from the beginning of school, this questionnaire is understandable for all Iranians and subsequently can be delegated to other cities [31]. Therefore, the result of this can be generalized in all part of Iran.

In order to evaluate the appropriate equivalence between the translated version and the original version, four stages of equivalence including semantic, idiomatic, experiential, and conceptual were considered [10]. The process of translation and cultural adaptation was completely performed regarding the four stages proposed by Herdman et al. [32].

Cronbach’s alpha correlation coefficient in the Persian version of CPQ11-14 ISF-16 was 0.855, which indicates sufficient internal reliability of the Persian version of the present questionnaire. This rate is similar to the results of studies performed in other countries. [2, 3, 5, 8, 11, 22, 23]

Participants were asked to complete the questionnaires in person and in a non-clinical setting where an acquainted assistant was present. We did not let the parents or guardians accompany the students since we alleged this would influence the results, [33] although the results of a study refuted the impact of parents’ responses on children while completing the OHRQoL questionnaire [34].

It should be noted that it might be possible that participants at this age-range become bored while filling out two questionnaires at the same time, and there will be a notion that this would lead them to fill out the questionnaires randomly and not precisely. However, the test–retest reliability results showed that the probability of random responses could be ignored [2]. Test–retest reliability in this study for the total scale was 0.85. This value for the original version of the 16-question form was 0.77 in Canada and in other studies, it was reported to be 0.78 in Saudi Arabia, 0.97 in Brazil, and 0.72 in Greece. [2, 5, 11, 23]

In this study, two dimensions of the Persian version of CPQ11-14 ISF-16 were extracted using Exploratory Factor Analysis (EFA). The first dimension, well-being, included questions 1, 2, 3, 4, 5, 6, 7, and 8, and the second dimension, symptom and function, included questions 9, 10, 11, 12, 13, 14, 15, 16, and 17. Similarly, in the study of Thomson et al. [3], two dimensions of CPQ11-14 ISF-16 questionnaire were extracted. However, in the original version of CPQ11-14 ISF-16, four dimensions were extracted. [5] Thomas reported that two eight-item subscales rather than four four-item ones might ensure greater statistical power and it can decrease probability of Type II error, principally where we have limitations on participant numbers, and the greater number of items grant us finer discrimination. [35]

Construct validity of the Persian version of CPQ11-14 ISF-16 was performed by evaluating the correlation between the average score of the two global questions and the total score and the average score of each of the dimensions of well-being and symptom and function. The results showed that there was a moderate correlation between these three items. These results were similar to the results presented by several studies. [3, 7, 11] However, the Arabic version did not report any correlation between well-being and symptom and function and global questions. [18]

To evaluate the criterion validity, the Persian version of CPQ11-14 ISF-16 was compared with the Persian version of the PIDAQ questionnaire. The validity and reliability of the Persian version of the PIDAQ questionnaire has already been evaluated in the Iranian population [25]. Moreover, the results showed a high correlation between PIDAQ questionnaire and CPQ11-14 ISF-16 questionnaire and two dimensions of well-being and symptom and function.

As the limitation of this study, the sample size was restricted due to COVID-19. Concerning the age of students, we preferred not to use electronic questionnaire so that we could be at hand to help students if any personal question or problem were present. Although pandemic pick had been ended during the study, teaching programs in most schools in Iran were still online and students were participating the school only in limited specifics days.

While the spoken Persian language varies between regions and countries, the written language has little differences. This study can be used as basic research for the next studies in countries in which people speak in Persian. Moreover, future studies performing in Iran, with larger sample sizes can exploit the results of the present study.

## Conclusion

The appraisal of the validity and reliability of the Persian version of the questionnaire CPQ11-14 ISF-16-GR verified it to be a suitable questionnaire for evaluating OHRQoL.

## Data Availability

The datasets used and/or analyzed during the present study are available from the corresponding author upon request.

## Abbreviations

WHO: World health organization
COHQoL: Child oral health quality of life
OHRQoL: Oral health quality of life
CPQ 11–14: Child perceptions questionnaire aged 11 to 14
PIDAQ: Psychosocial impact of dental aesthetics questionnaire
ANOVA: Analysis of variance
EFA: Exploratory factor analysis
